# Emerging Trends and Research Foci in Cataract Genes: A Bibliometric and Visualized Study

**DOI:** 10.3389/fgene.2021.610728

**Published:** 2021-08-09

**Authors:** Hongli Zhu, Zhichang Zhang

**Affiliations:** ^1^Department of Ophthalmology, The 4th People's Hospital of Shenyang, Shenyang, China; ^2^Department of Computer, School of Intelligent Medicine, China Medical University, Shenyang, China

**Keywords:** cataract, gene, data visualization, bibliometric, citespace, VOSviewer

## Abstract

**Background:** Approximately 50% of cataracts are associated with genetic factors. Genetic etiology and molecular mechanisms based on gene research increase the understanding of cataracts and provide direction for diagnosis and intervention. In the present study, SCIE papers related to the modeling of cataract gene research from 2010–2019 were evaluated and qualitative and quantitative analyses with modeling performed.

**Methods:** The SCIE database was searched on July 6, 2021 for cataract gene publications and relevant papers published since 2010 were considered for review. Subsequently, 1,904 SCIE papers associated with cataract genes from 2010–2019 were analyzed using a bibliometric method. The publication, country, institution, journal, references, knowledgebase, keywords, and research hotspots of the papers were analyzed using an online analysis platform of literature metrology, bibliographic item co-occurrence matrix builder (BICOMB), CiteSpace V, and VOS viewer analysis tool.

**Results:** 78 countries published the related articles, and the United States ranks of America had the most publications. Two thousand seven hundred and eighty three institutions contributed to the related publications. Fudan University had the most publications. The reference clusters of SCI papers were clustered into six categories, namely, causing congenital cataract-microcornea syndrome, functional snp, cataractous lenses, a1 mutation, foxe3 mutation, cell adhesion gene pvrl3, nid1 gene. The key words representing the research frontiers were cerebrotendinous xanthomatosis (2017-2019), oxidative stress (2017–2019).

**Conclusion:** This study provided a systematic, objective and comprehensive analysis of the literature related to gene research of cataract. Moreover, this study demonstrated the current hotspots and the future trends in the field of gene research of cataract. This review will help ophthalmologist to discern the dynamic evolution of cataract gene research, as well as highlight areas for future research.

## Background

Cataract is not only a multifactorial but also a monogenic disease. In addition to genetic factor, its pathogenesis and development are also related to age, gender, radiation, oxidation, physical injury, diet, and medication. These factors can lead to abnormal gene expression and affect the transparency of the lens, and eventually form cataract. Despite the in-depth study of eye's genes, It is still hard to find many causes that lead to abnormal lens (Gillespie et al., 2014). Hereditary cataract is a kind of clinical and genetic heterogeneity disease (Berry et al., 1999). Isolated cataract and syndromic congenital cataract are a heterogeneous developmental defect. The identification of the related genes is challenging (Anand et al., 2018b). After overcoming the bottleneck of genetic heterogeneity, more and more people realize that the explanation of genetic variation and the relationship between new genes and specific phenotypes are still challenging. However, further understanding of the heredity and variation basis of lens and anterior segment abnormalities will be of great value to our understanding of eye diseases.

Genetic studies have identified mutations in over 30 causative genes for congenital or other early-onset forms of cataract as well as several gene variants associated with age-related cataract (Shiels and Hejtmancik, 2017). Cataract is a major cause of blindness worldwide. It is characterized by lens opacification and is accompanied by extensive post-translational modifications (PTMs) in various proteins (Zhang et al., 2018). PTMs play an essential role in lens opacification. And post-translational modification (PTM) of lens proteins is believed to play various roles in age-related lens function and development. Several PTMs have been described in proteins isolated from relatively old human lenses, including phosphorylation, deamidation, racemization, truncation, acetylation, and methylation. An overwhelming majority of previous cataract proteomic studies have exclusively focused on crystallin proteins, which are the most abundant proteome components of the lens. Elucidating the role of these modifications in cataract formation has been a challenging task because they are among the most difficult PTMs to study analytically (Huang et al., 2011). The proteomic status of some amides presents similar properties in normal aged and cataractous lenses, whereas some may undergo greater PTMs in cataract.

In this study, conducted from Jan 1, 2010 to Dec 31, 2019, we analyzed the SCIE papers for studies related to cataract gene research using bibliometric methods, and included articles on cataract research published in various countries, regions, and by different research institutions. We further analyzed journals that published papers on cataract research, and we analyzed the “top 10 cited references,” and we calculated the number of times popular references were cited. By clustering the reference network of co-cited references, we also analyzed the knowledge base of this topic. The research hotspots of this topic were detected by burst keywords, which could provide some reference for future relevant research (Small, 1973; Chen, 2006). These analyses afford ophthalmologist with both a macroscopically understanding and a microscopically characterization of the knowledge domain as a whole. Compared with traditional systematic reviews written by experts, this bibliometric analysis provides a timely, visual, and unbiased approach to track the development and explore the specific knowledge domains.

## Methods

**Figure 1:**
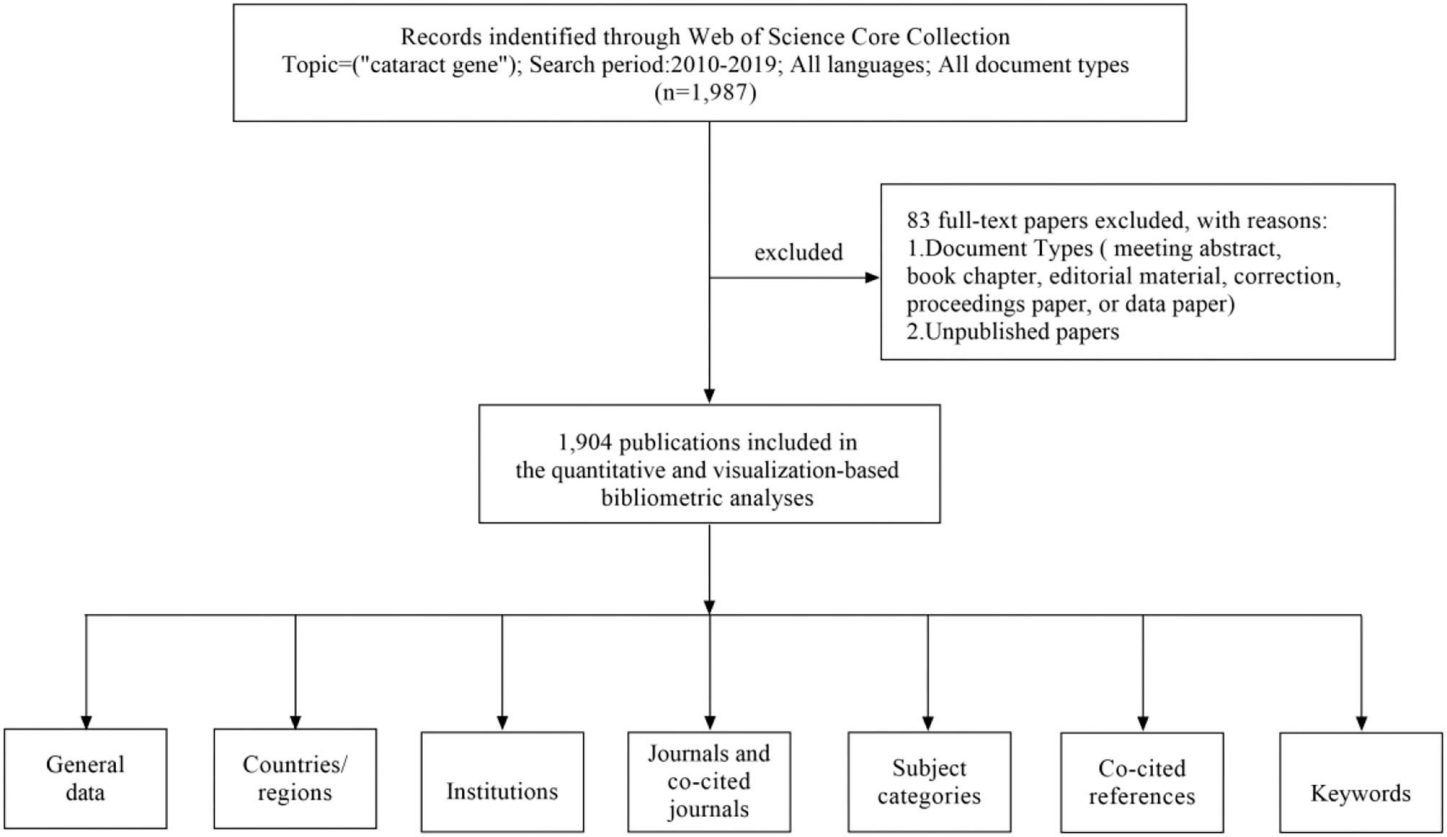
A frame flow diagram. The diagram showed details selection criteria for Cataract gene publications from WoSCC database and the steps of bibliometric analysis.

All data were downloaded from Web of Science Core Collection (WoSCC) on July 6, 2021, and were verified by two authors (ZH and ZZ) independently. The literature research was performed for publications from 2010 to 2019, using the following search terms (TS = cataract^*^ and TS =gene), and The literature types was all document types. We collected the following basic information for each article: title, abstract, authors, institution, country/region, journal, keywords, and references. Articles that met the following criteria were included: (1) those indexed in the Web of Science Core Collection and (2) The following articles were excluded: (1) irrelevant meeting abstracts, irrelevant proceedings paper, book chapter, data paper, editorial material, and repeated articles and (2) unpublished documents without enough information for further analysis. A total of 83 papers with duplicates were excluded. The detailed search processes and analysis procedures were shown in Figure 1.

## Data Analysis

We tried to describe all publication characteristics, including countries, institutes, journals, keywords, and so on. We inquired the H-index, which was regarded as an important indicator to measure the scientific value of research (Eyre-Walker and Stoletzki, 2013). In this study, the Online Analysis Platform of Literature Metrology (http://bibliometric.com/), CiteSpace V (Drexel University, Philadelphia, PA, USA) and VOSviewer (Leiden University, Leiden, the Netherlands) were used to perform co-occurrence analysis and visualize the collaborative networks of the countries/institutes/journals/keywords. Through CiteSpace, reference co-citation analysis was performed, and related knowledge maps were constructed, and burst keyword detection was also performed to investigate the recurrent new keywords (Chen, 2006).

## Results

### Distribution of Articles by Publication Years

**Figure 2:**
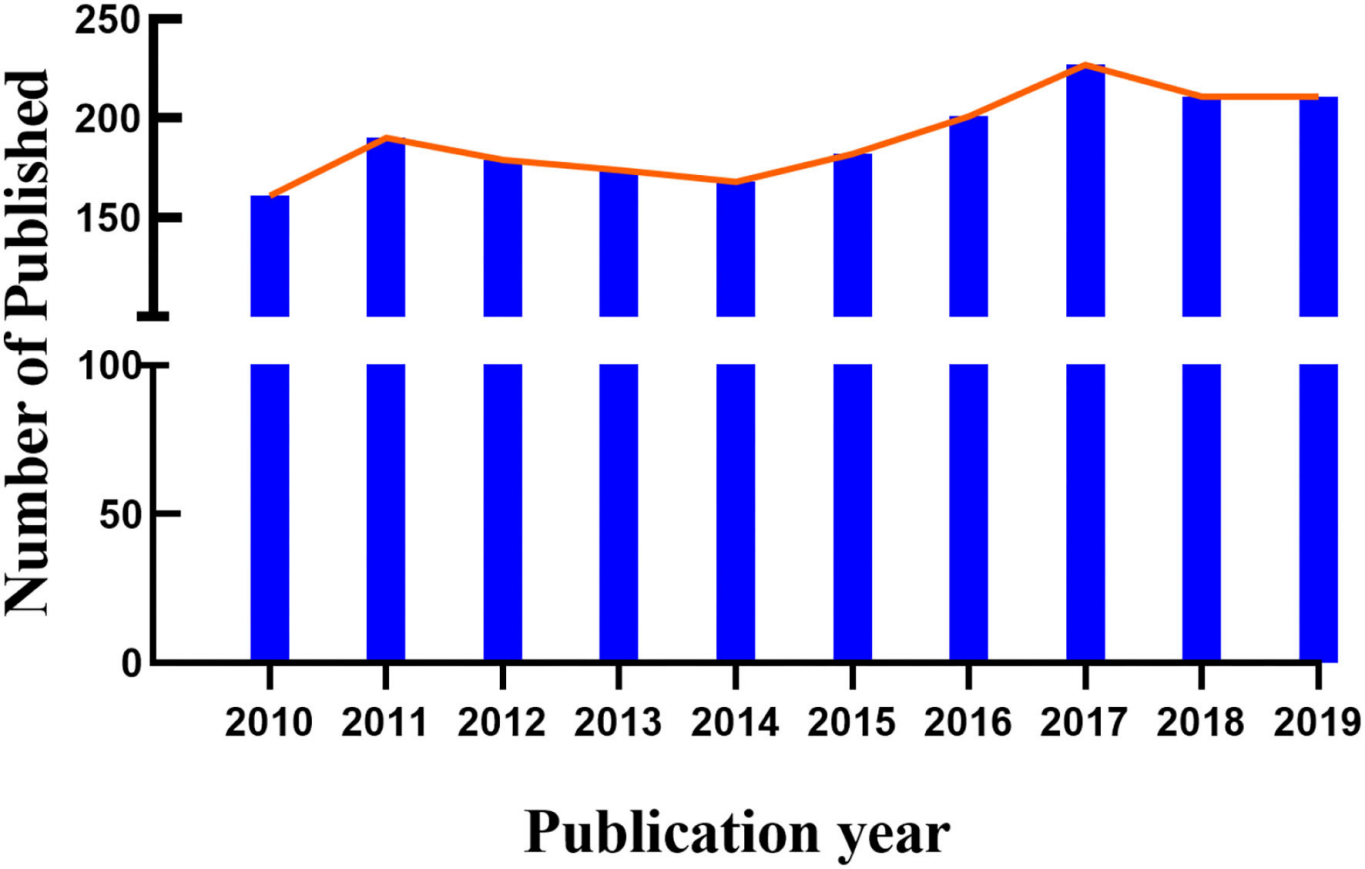
Trends in the number of publications on Cataract-gene from 2010 to 2019.

A total of 1904 papers from 2010 to 2019 were published. Figure 2 shows the trend in the number of cataract gene related publications. Since 2017, the activity in cataract gene research reached a peak.

### Countries/Regions and Institutes

**Figure 3:**
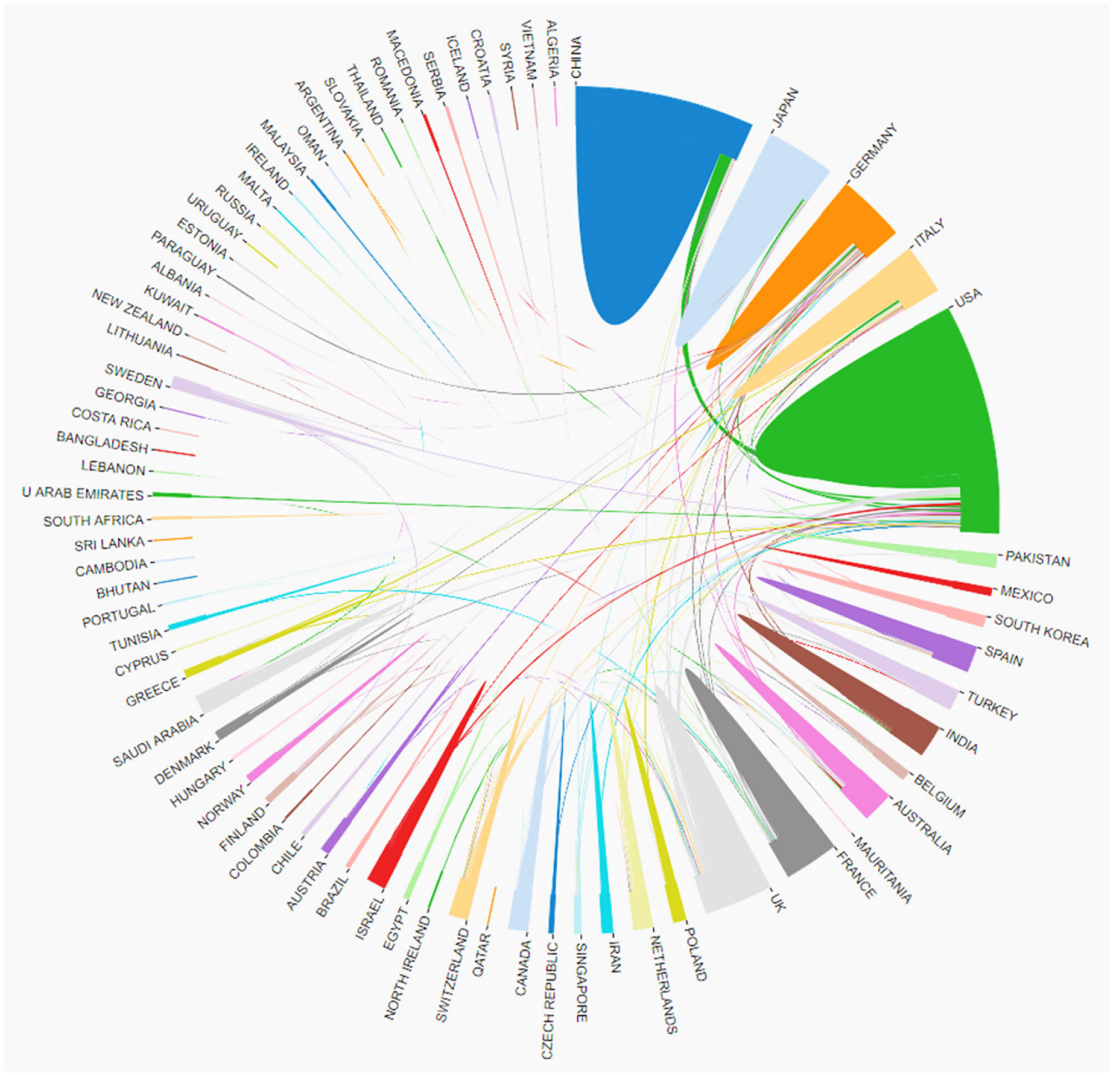
The cooperation of countries/regions contributed to publications on Cataract-gene from 2010 to 2019.

A total of 78 countries / regions published the related articles, Collaborations among these countries were shown in Figure 3. The top 10 countries were listed in Table 1. The United States of America (USA) had the most publications (581), followed by China (487), Germany (160) and the United Kingdom (146).

**Table 1:** The top 10 Countries/Regions and Institutions to publications on Cataract-gene from 2010 to 2019.

**Rank**	**Countries/regions**	**Count**	**H-index**	**Institutions**	**Count**	**H-index**
1	USA	581	46	FUDAN UNIV	52	14
2	PEOPLES R CHINA	487	28	SUN YAT SEN UNIV	45	11
3	GERMANY	160	31	NEI	42	16
4	UK	146	28	CAPITAL MED UNIV	33	12
5	JAPAN	138	23	WASHINGTON UNIV	32	11
6	INDIA	104	18	ZHEJIANG UNIV	31	14
7	FRANCE	95	24	CENT S UNIV	30	15
8	ITALY	103	23	JOHNS HOPKINS UNIV	30	7
9	CANADA	70	21	CHINESE ACAD MED SCI	27	14
10	AUSTRALIA	67	22	HARVARD UNIV	26	12

**Figure 4:**
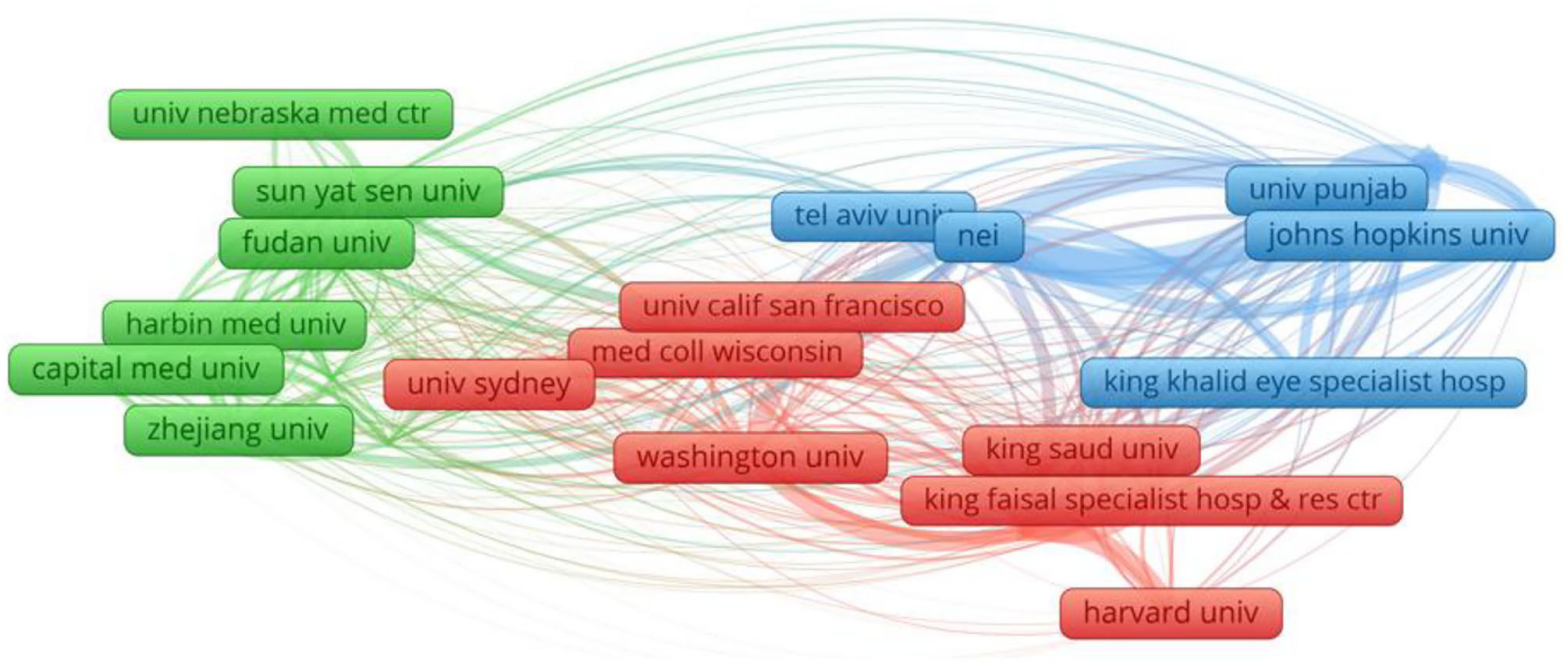
Network map of institutions contributed to publications on Cataract-gene from 2010 to 2019.

A total of 2,783 institutes contributed to the related publications, the top 10 institutes listed in Table 1. Collaborations among these institutes were shown in Figure 4. Fudan University had the most publications (52), followed by Sun Yat Sen University (45), the National Eye Institute (42), and Capital Med University (33).

### Journals

**Figure 5:**
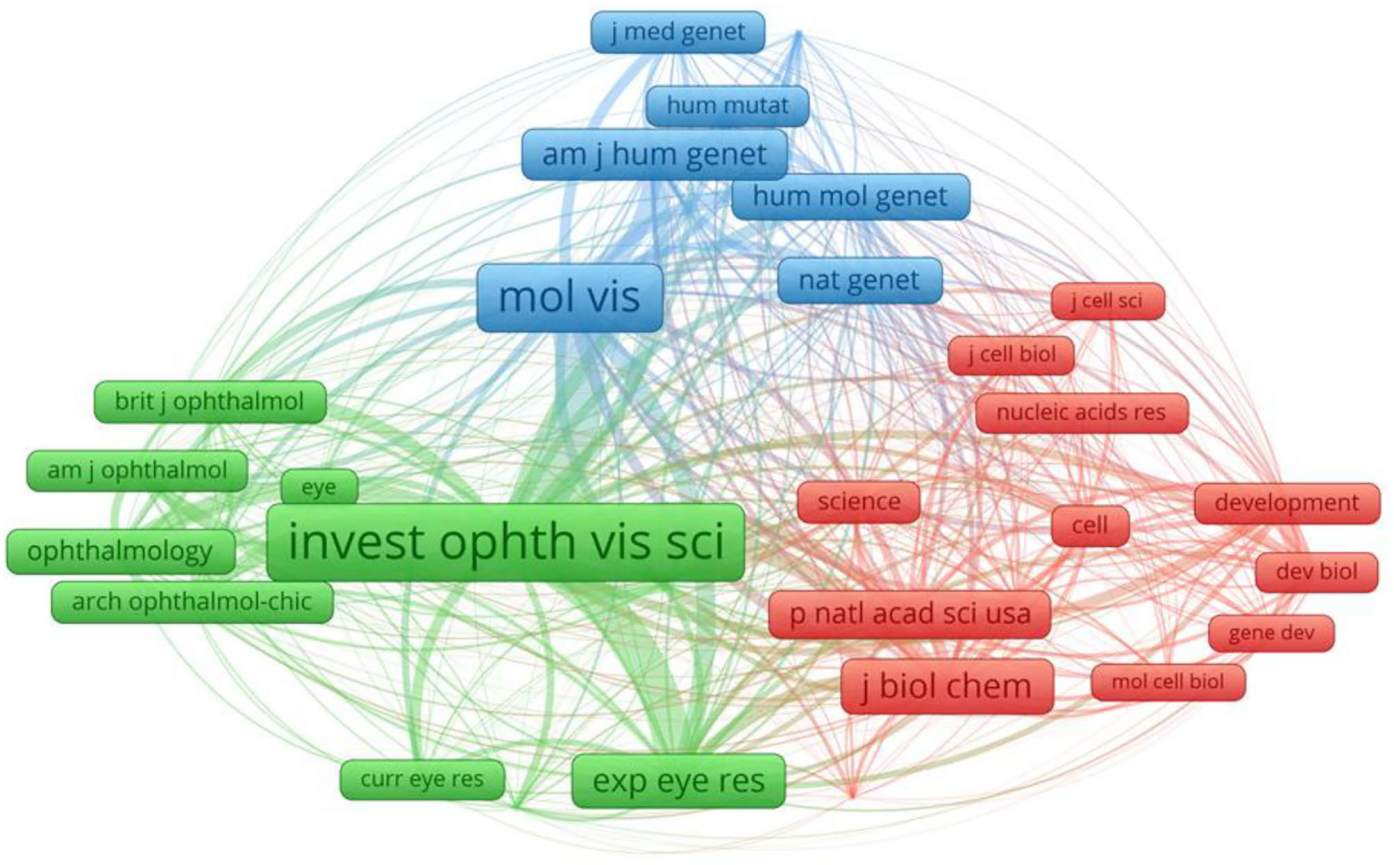
The network map of cited journals contributed to publications on Cataract-gene from 2010 to 2019.

The referential relationship of academic journals represents the situation of knowledge exchange in the research field in question, where the citing papers form the frontier of knowledge, and the cited papers the knowledge basis. The top 10 journals were presented in Table 2. Collaborations among these journals were shown in Figure 5. The first was MOL VIS (151), followed by INVEST OPHTH VIS SCI (92).

**Table 2:** The top 10 journals and references of publications on Cataract-gene from 2010 to 2019.

**Rank**	**Source titles**	**Count**	**Title of co-cited reference**	**Pmid**	**Count**	**Interpretation of the findings**
1	MOL VIS	151	Congenital cataracts and their molecular genetics (Hejtmancik, 2008)	18035564	91	This study reported the molecular genetics of congenital cataracts.
2	INVEST OPHTH VIS SCI	92	Cat-Map: Putting Cataract on the Map (Shiels et al., 2010)	21042563	77	This paper summarized the genetic complexity of Mendelian and age-related cataract by Cat-Map.
3	PLOS ONE	89	Genetics of Human Cataract (Shiels and Hejtmancik, 2013)	23647473	42	This study showed the genetics associated with cataract.
4	OPHTHALMIC GENET	47	A Method and Server for Predicting Damaging Missense Mutations (Adzhubei et al., 2010)	20354512	37	This article presented PolyPhen-2 software to analyze the effects of non-synonymous mutations on proteins in humans
5	CURR EYE RES	42	Molecular Characteristics of Inherited Congenital Cataracts (Huang and He, 2010)	20624502	33	This paper showed the characteristics of inherited congenital cataracts.
6	EXP EYE RES	42	Comprehensive mutational screening in a cohort of Danish families with hereditary congenital cataract (Hansen et al., 2009)	19182255	32	This study analyzed family cohorts with inherited congenital cataract in a comprehensive mutation screening strategy and demonstrated the effectiveness of the strategy.
7	AM J MED GENET A	41	Crystallin gene mutations in Indian families with inherited pediatric cataract (Devi et al., 2008)	18587492	32	This document identified the frequency and frequency of crystalloprotein gene mutations in a population of Indian cataract patients
8	INT J OPHTHALMOL-CHI	28	The EPHA2 Gene Is Associated With Cataracts Linked to Chromosome 1p (Shiels et al., 2008)	19005574	29	This article provided the EPHA2 gene is associated with cataracts Linked to chromosome 1p.
9	SCI REP-UK	27	Mutation analysis of CRYAA, CRYGC, and CRYGD associated with autosomal dominant congenital cataract in Brazilian families (Santana et al., 2009)	19390652	29	This study reported the results of an analysis of mutations in CRYAA, CRYGC, and CRYGD associated with autosomal dominant congenital cataracts.
10	AM J HUM GENET	23	Mutations in the RNA granule component TDRD7 cause cataract and glaucoma (Lachke et al., 2011)	21436445	28	This article identified that TDRD7-RGs play an essential role in the regulation of specific genes that are critical for lens development.

**Figure 6:**
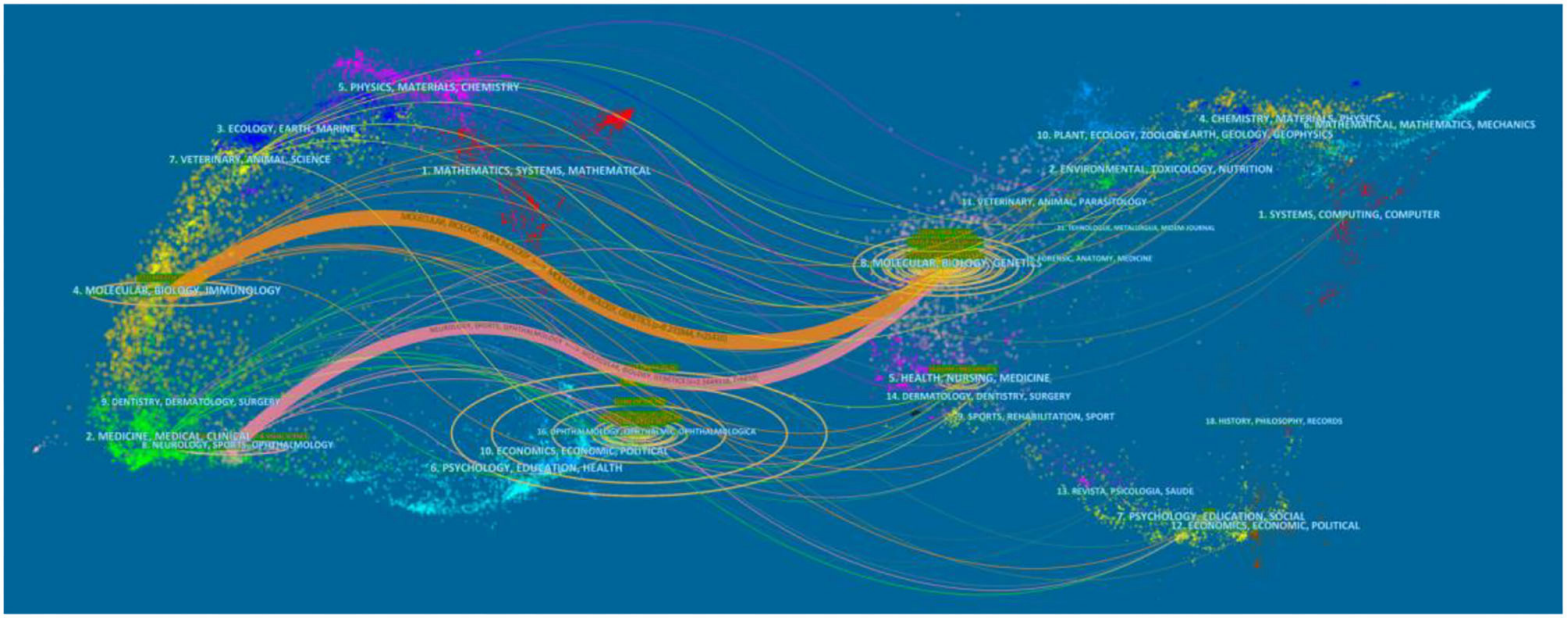
The dual-map overlay of journals contributed to publications on Cataract-gene from 2010 to 2019.

The dual-map overlay of journals is shown in Figure 6, with the citing journals on the left side, cited journals on the right side, and the colored paths indicate the citation relationships. The color paths indicated that studies, published in Molecular/Biology/Genetics journals, are usually cited in the studies, published in Molecular/Biology/Immunology and Neurology/Sports/Ophthalmology journals.

### References

Analysis of references is one of the most important indicators of bibliometric. Frequently cited documents are generally of great influence in their respective research field. On this topic, a co-cited documents-based clustering analysis may present subfields and connecting nodes of the research in question.

**Figure 7:**
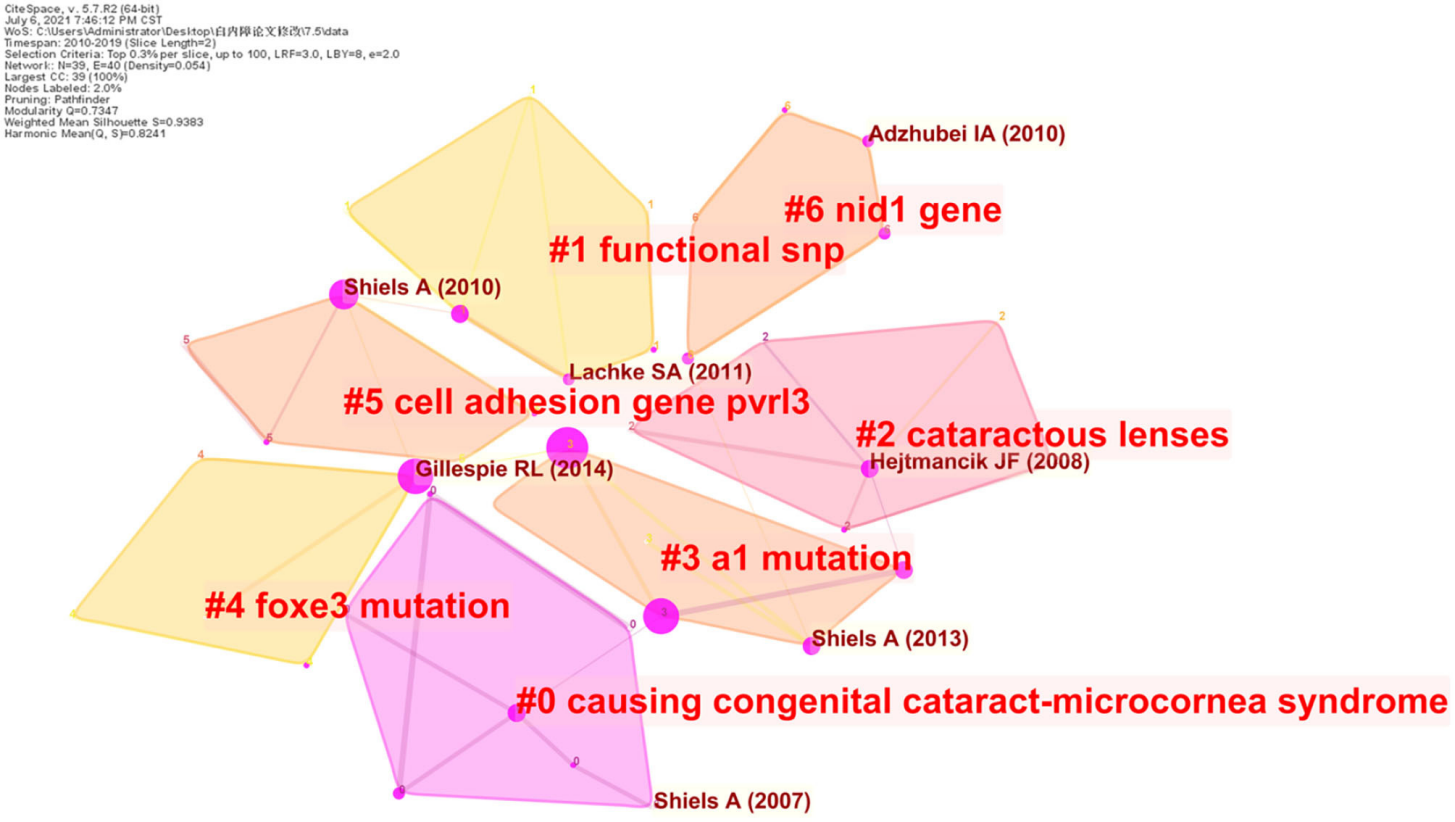
Reference co-citation map of publications on Cataract-gene from 2010 to 2019.

A network of co-cited references was constructed to assay the scientific relevance of the related publications (Figure 7). The cluster setting parameters were as follows: # Years Per Slice = 2, Top N% = 0.3, pruning algorithm was adopted. The Modularity Q score was 0.7347, >0.5, showing the network was reasonably divided into loosely coupled clusters. The Weighted Mean silhouette score was 0.9383, more than 0.5, indicating that the homogeneity of these clusters was acceptable. Index items extracted from literature were used as cluster markers. The largest cluster #0 was marked as “causing congenital cataract-microcornea syndrome” (Huang and He, 2010; Hu et al., 2010; Jiang, 2010; Li et al., 2010) the next cluster #1 was marked as “functional snp”(Gu et al., 2016; Wang et al., 2016; Anand et al., 2018a), the second cluster #2 was marked as “cataractous lenses” (Sousounis and Tsonis, 2012; Weisschuh et al., 2012; Deng and Yuan, 2014), the third cluster #3 was marked as “a1 mutation” (Zhu et al., 2010; Yu et al., 2012; Wu et al., 2017), the fourth cluster #4 was marked as “foxe3 mutation” (Wada et al., 2011; Anand et al., 2018a; Plaisancie et al., 2018), cluster #5 was marked as “cell adhesion gene pvrl3” (Lachke et al., 2012; Weatherbee et al., 2019), cluster #6 was marked as “nid1 gene” (Murgiano et al., 2014; Osinchuk et al., 2017; Braun et al., 2019).

### Keywords

**Figure 8:**
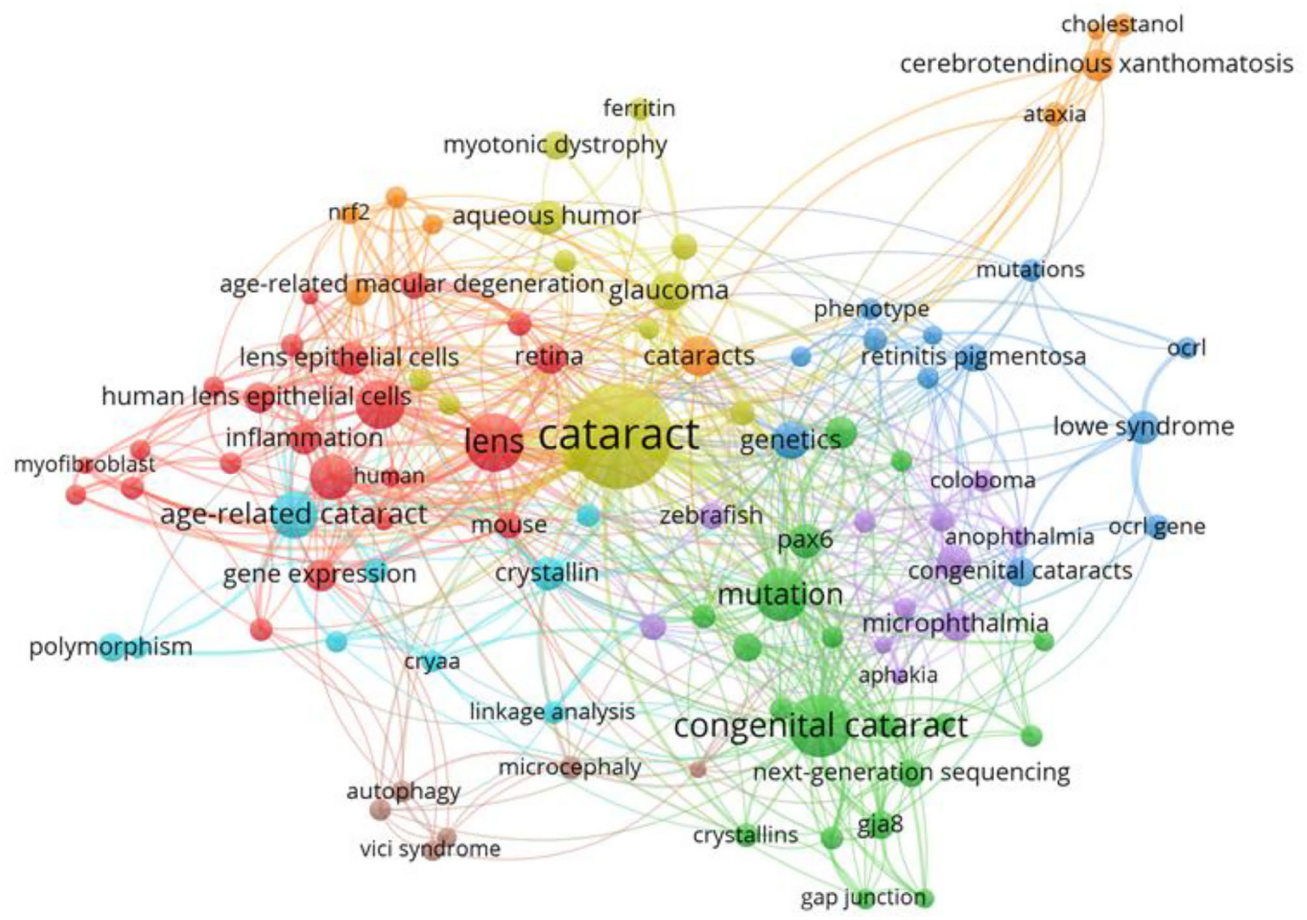
The network map of keywords showed 58 keywords and divided into 5 clusters.

**Figure 9:**
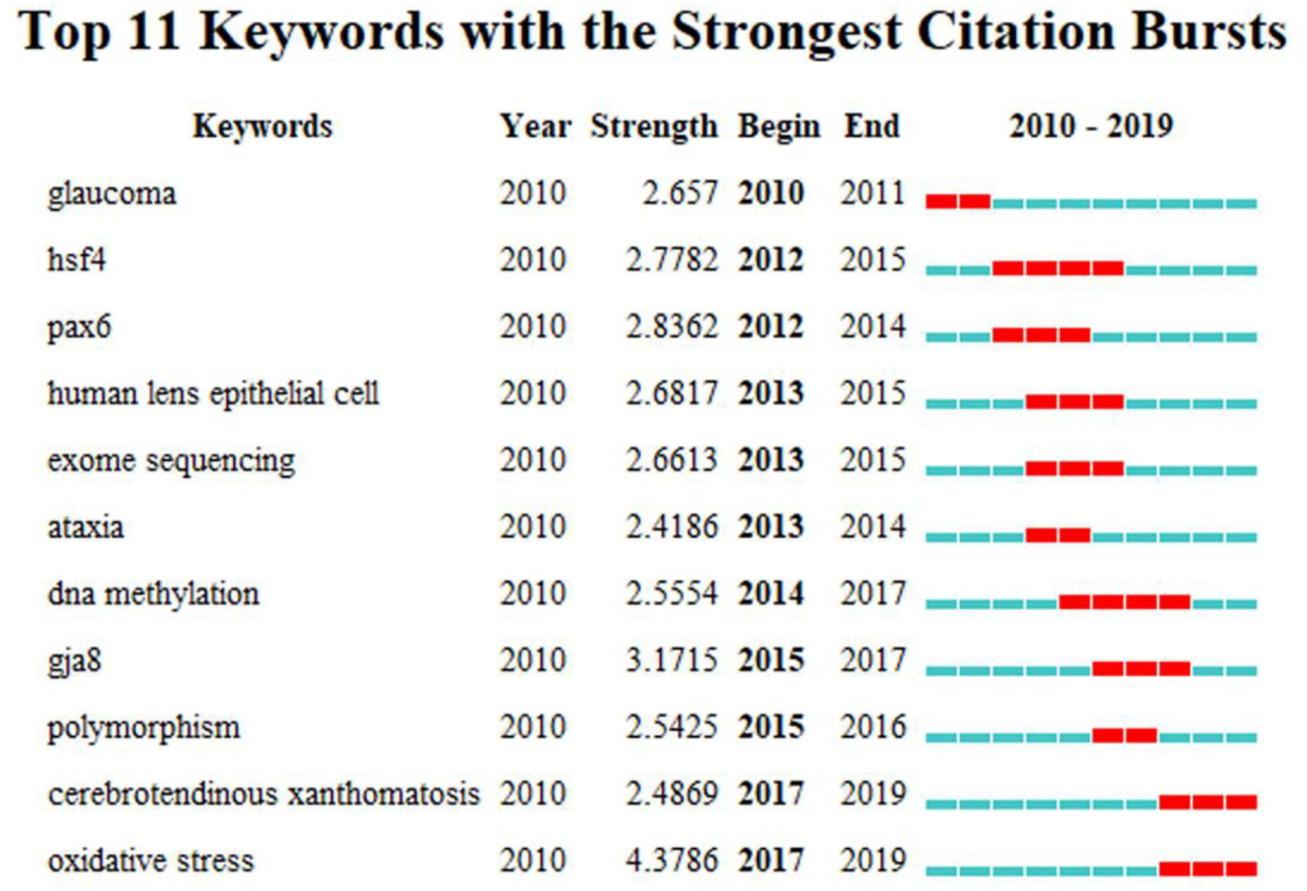
The keywords with The strongest citation bursts of publications on Cataract-gene from 2010 to 2019.

Keywords in the related publications were extracted and analyzed. The top 20 keywords were listed in Table 3. In addition to cataract, congenital cataract and mutation occurred more than 50 times. Keywords analysis of the 1,904 articles identified 100 keywords with a minimum of 20 occurrences and divided them into five clusters (cataract, gene, mutation, age-related cataract, differentiation) (Figure 8). We analyzed the temporal trend of hotspot shift according to the top 11 keywords with the strongest citation bursts, such as cerebrotendinous xanthomatosis (2017–2019), and oxidative stress (2017–2019) (Figure 9).

**Table 3:** The top 20 Keywords on Cataract-gene from 2010 to 2019.

**Rank**	**Keyword**	**Count**	**Rank**	**Keyword**	**Count**
1	Cataract	147	11	Gene expression	12
2	Congenital cataract	72	12	Apoptosis	12
3	Mutation	50	13	Lowe syndrome	11
4	Len	34	14	pax6	11
5	Age-related cataract	24	15	gja8	9
6	Exome sequencing	16	16	Microphthalmia	8
7	Genetics	15	17	Next-generation sequencing	8
8	Oxidative stress	15	18	Linkage	7
9	Aniridia	15	19	Anophthalmia	7
10	Crystallin	14	20	Retinitis pigmentosa	6

## Discussion

### General Data

One thousand nine hundred and four SCI papers related to the gene research of cataract and published from 2010 to 2019 were analyzed in this study. The United States of America had the most publications (581), accounting for 30.9%. China had the second most publications (487), accounting for 27.1%. The top 10 institutions included three in the United States of America, six in Asia, and one in Australia. MOL VIS was the most published journal. These observations showed that MOL VIS predominantly contributed to the research in this field. In addition, the top 10 cited publications were also investigated. The first article was published in SEMINARS IN CELL & DEVELOPMENTAL BIOLOGY by HEJTMANCIK JF, and was cited 91 times. The second was published in MOLECULAR VISION by SHIELS A, which was cited 77 times.

### Knowledge Base

According to previous studies, many genetic types of cataract have been investigated in animal models. Substantial advances have taken place regarding the mapping of genes and their variations involved in congenital cataract formation, and the genetic causes of age-related cataract have been discovered. As shown in Figure 6, after clustering the co-cited references, the key nodes in the clustering resulted reveal the knowledge bases in this research field. Namely: #0 “causing congenital cataract-microcornea syndrome,” #1 “functional snp,” #2 “cataractous lenses,” #3 “a1 mutation,” #4 “foxe3 mutation,” #5 “cell adhesion gene pvrl3,” #6 “nid1 gene.” This paper described the knowledge base of cataract gene research according to different clusters.

In #0 “causing congenital cataract-microcornea syndrome,” Shanshan Hu et al. identified the underlying genetic defect in a four-generation family of Chinese origin with autosomal dominant congenital cataract-microcornea syndrome (CCMC). They direct sequencing of the encoding regions of the candidate genes revealed a heterozygous mutation c.592CT in exon 2 of the gap junction protein, alpha8 (GJA8) gene. This mutation was responsible for the familial disorder through the substitution of a highly conserved arginine to tryptophan at coden198 (p.R198W). That report is the first to relate p.R198W mutation in GJA8 with CCMC. The result expanded the mutation spectrum of GJA8 in associated with congenital cataract and microcornea and implied that this gene had direct involvement with the development of the lens as well as the other anterior segment of the eye.

In #1 “functional snp,” SNPs is single-nucleotide polymorphisms. As DNA repair is implicated in ARC pathogenesis and SNPs in the 3′-terminal untranslated region (3′-UTR) targeted by micro RNA(miRNAs) can alter the gene function.

In #2 “cataractous lenses,” Konstantinos Sousounis et al. had examined the patterns of gene expression in cataractous lenses. The purpose was to evaluate unique and common patterns of gene expression during development, aging and cataracts.

In #3 “a1 mutation,” Yanan Zhu et al. first report of a phenotype of progressive nuclear and cortical cataracts related to the βA1/A3-crystallin gene (CRYBA3/A1) mutation IVS3+1 G>A. This finding expands the spectrum of cataract phenotypes caused by the IVS3+1 G>A mutation of CRYBA3/A1, confirms the phenotypic heterogeneity of this mutation and suggests the mechanism that influences the cataractogenesis in different ethnic backgrounds. C.30-2 A>G mutation of CRYBA3/A1 gene is a novel mutation and broadens the genetic spectrum of ADCC.

In #4 “foxe3 mutation,” FOXE3 gene, which was initially described in individuals with dominantly inherited anterior segment dysgenesis and, subsequently, associated with recessively inherited primary aphakia, sclerocornea and microphthalmia. Mutations in the transcription factor genes FOXE3 cause congenital lens defects including cataracts that may be accompanied by defects in other components of the eye or in nonocular tissues. All individuals with ocular abnormalities described in the literature for which a FOXE3 mutation was identified and demonstrated that correlations exist between the mutation type, mode of inheritance and the phenotype severity. a mutation was located in the regulatory regions of the Foxe3 gene. This gene is responsible for cataracts in humans and mice, and it plays a crucial role in the development of the lens. Furthermore, mutation of Foxe3 causes various ocular defects. Kenta Wada et al. suggested that cataracts in rct mice were caused by reduced Foxe3 expression in the lens and that this decreased expression was a result of a deletion in a cis-acting regulatory element. Deepti Anand et al. comprehensively describe here all the variants in FOXE3 genes linked to human developmental defects. A total of 52 variants for FOXE3, the effort revealed FOXE3 had 33 unique causal mutations. Finally, they made the detailed FOXE3 variant information available in the Leiden Online Variation Database (LOVD) platform at https://www.LOVD.nl/FOXE3.

In #5 “cell adhesion gene pvrl3,” the expression of PVRL3, which encodes the cell adhesion protein Nectin 3, is significantly reduced in patient DGAP113 lymphoblastoid cells, likely due to a position effect caused by the chromosomal translocation. Moreover, Pvrl3 knockout mice as well as a spontaneous mouse mutant ari (anterior retinal inversion), that maps to the Pvrl3 locus, exhibit lens and other ocular defects involving the ciliary body. Collectively, these data identify PVRL3 as a critical gene involved in a Nectin-mediated cell-cell adhesion mechanism in human ocular development.

In #6 “nid1 gene,” nidogen 1 (NID1) gene (c.3579_3604+829del) deletion leads to the skipping of exon 19 during transcription and is therefore predicted to cause a frameshift and premature stop codon (p.1164fs27X). Nidogen 1 deficient mice show neurological abnormalities and highly irregular crystal lens alterations. This study adds NID1 to the list of candidate genes for inherited cataract in humans and is the first report of a naturally occurring mutation leading to non-syndromic cataract in cattle provides a potential large animal model for human cataract.

### Research Hotspots and Frontiers

Keywords concentrate expression of current research issues or concepts. Burst keywords stand for emerging trends and research frontiers. In the present study, We further used CiteSpace to capture the burst keywords. Two frontiers of related research were found as follows: cerebrotendinous xanthomatosis (2017–2019) (Tibrewal et al., 2017; Freedman et al., 2019), oxidative stress (2017–2019) (Zoric et al., 2008; Petrou and Terzidaki, 2017), and these key words cover the research frontier of the current topic.

#### Cerebrotendinous Xanthomatosis

CTX is a uncommon autosomal recessive metabolic condition, which is characterized by multiple system damage caused by lipid metabolism disorders, and is often manifested as tenoxanthoma. In cataract in adolescence and arteriosclerosis in early years, cholestanol and other metabolites are often deposited in lipid-rich nerve tissue, which can cause neurotoxicity and lead to damage of central and peripheral nerves. The pathogenic gene of CTX is located at 2q33-qter. The variation of the CTX gene causes the deficiency of CYP27, resulting in cholesterol metabolism disturbance and the accumulation of cholesterol, cholestanol and other neurotoxic substances in various tissues and organs, particularly nervous tissue. Freedman et al. studied the prevalence of the CTX gene in a study population that was diagnosed with idiopathic, early-onset, and bilateral cataracts. The study included patients aged 2–21 years; 1.8% of these patients were diagnosed as having the CTX gene variation. Within this study population, the CTX gene was about 500-fold the presently estimated its prevalence in human (3 to 5/100 000). The data suggested that idiopathic, early-onset, and bilateral cataracts could be a screening tool for CTX for early identification (Freedman et al., 2019). Tibrewal et al. previously reported the case of a child who presented with bilateral cataracts, which led to a diagnosis of CTX. They described the cataracts' morphologic characteristics and the outcome of systemic treatment on cataract. The authors believed CTX to be an uncommon autosomal recessive disease that results in lipid storage abnormalities and presents in a wide range of clinical manifestations, including juvenile bilateral cataracts. Left untreated, CTX can lead to irreversible progressive neurologic devastation and early death. Frequently, juvenile bilateral cataracts occur in early childhood; this allows ophthalmologists the chance to make an early diagnosis and initiate the sysmetic treatment (Tibrewal et al., 2017).

#### Oxidative Stress

*In vivo*, oxidative stress is a state of imbalance between oxidation and antioxidation; this state results in inflammatory infiltration of neutrophils, an increase in protease secretion, and a production of numerous oxidation intermediates. Oxidative stress has a negative effect on the body as a result of free radicals and is considered to be an important factor leading to aging and disease. Zoric et al. reviewed a retrospective cross-sectional study that evaluated 80 samples of aqueous humor and corticonuclear lens blocks. The authors believed that cataract type and pigmentation may be dictated by the form and intensity of oxidative stress. If true, this would make efforts in cataract prevention challenging and more complex. Zoric et al. proposed that the role of oxidative stress in cataract formation was not the same for all cataract types. For example, high levels of lipid peroxides may result in certain pigmented cataracts whereas lipid peroxidation and consumption of SH groups (as seen in the development of cortical cataracts) might be of less importance (Zoric et al., 2008). Petrou et al. calculated the thermodynamic parameters for nucleation, elongation, fibrillization, and other processes of proteinaceous diseases that were related to β-amyloid protein (Alzheimer disease), tau protein (Alzheimer and Pick disease), α-synuclein (Parkinson disease), prion, amylin (type 2 diabetes), and α-crystallin (cataract). From kinetic data (k, T), it can be concluded that ΔG≠ is equal to the energy needed for ground state oxygen excitation of the singlet oxygen state (1Δg, first excited). The similarity of ΔG≠ values is an indication that there may be a common mechanism in the previously mentioned disorders. Petrou and colleagues attributed this common mechanism to oxidative stress and specifically to the singlet oxygen molecule (1Δg) (Petrou and Terzidaki, 2017).

## Conclusion

Using the bibliometric analysis this study provided a systematic analysis of the literature related to cataract gene. The analysis was also objective and comprehensive. Moreover, this study demonstrated the research basis, the current hotspots and the future trends in the field of cataract gene. The knowledge bases in this research field were causing congenital cataract-microcornea syndrome, functional snp, cataractous lenses, a1 mutation, foxe3 mutation, cell adhesion gene pvrl3, nid1 gene. The emerging trends and research frontiers of current research theme were cerebrotendinous xanthomatosis and oxidative stress. Papers published at different stages were collected for this study, some of which were not comprehensive and might have publication bias, which may affect the results of this systematic review.

## Data Availability Statement

The raw data supporting the conclusions of this article will be made available by the authors, without undue reservation.

## Author Contributions

HZ and ZZ designed and conceived the general idea and context of this review, wrote and integrated all sections, and contributed to the relevant references of this manuscript. All the authors read and approved the final manuscript.

## Conflict of Interest

The authors declare that the research was conducted in the absence of any commercial or financial relationships that could be construed as a potential conflict of interest.

## Publisher's Note

All claims expressed in this article are solely those of the authors and do not necessarily represent those of their affiliated organizations, or those of the publisher, the editors and the reviewers. Any product that may be evaluated in this article, or claim that may be made by its manufacturer, is not guaranteed or endorsed by the publisher.

## Abbreviations

BICOMB: bibliographic item co-occurrence matrix builder
CTX: cerebrotendinou xanthomatosis
WoSCC: Web of Science Core Collection
TRPM3: transient receptor potential cation channel subfamily M member-3
LOD: logarithm of odds
EPHA2: Eph-receptor type-A2
ADCC: autosomal dominant congenital cataract.
